# Duchenne muscular dystrophy involves the myocardium and causes arrhythmia: Case report

**DOI:** 10.3389/fcvm.2022.974843

**Published:** 2022-11-09

**Authors:** Xuhan Liu, Wei Zhao, Shangzhi Shu, Weihua Zhang

**Affiliations:** Department of Cardiovascular Medicine, The First Hospital of Jilin University, Changchun, China

**Keywords:** heart failure, arrhythmia, duchenne and baker muscular dystrophies, myocardial damage, ARNI

## Abstract

**Background:**

Patients with muscular dystrophy have mutations in the gene that can lead to severe muscle wasting, respiratory issues or heart failure between ages 30 and 40. Currently, there is no effective treatment for DMD-induced heart failure.

**Case presentation:**

We report a patient with recurrent unexplained fever and muscle soreness was definitely diagnosed with DMD. An analysis of the patient's genetics revealed a nonsense mutation (C.1207G > T). His DMD was treated with hormones. Also, the patient's fever is under control because of hormone therapy. However, as the disease progresses, the heart structure and function gradually change, and eventually malignant arrhythmias occur.

**Conclusion:**

We report a rare case of DMD involving the heart causing heart failure and malignant arrhythmia. Currently, no complete treatment is available for these patients, but our treatment regimen may benefit our patient and improve his outcomes.

## Background

Duchenne Muscular Dystrophy (DMD) is genetic or caused by mutations of motor function proteins (1). It is often diagnosed in childhood, contributing to life-long disabilities and shorter lifespans (1). Mutations of the Duchenne gene, located on chromosome Xp21.2, are the cause of most Duchenne muscular dystrophies in children (2). Mutations in the dystrophin gene lead to serious muscle wasting, respiratory or cardiac failure by the age of 30 (3). It is estimated to be 1/5,000 in the population (1). The disease progresses rapidly, with wheelchair dependence occurring in the teens (4) and usually progresses to a severe cardiomyopathic condition. Here we present a case of a patient with DMD and a nonsense mutation (C.1207G > T), faced intermittent fever and muscle pain all over the body repeatedly that eventually advanced to heart failure and arrhythmia.

## Case presentation

A 21-year-old Chinese man was diagnosed in June 2002 with muscular dystrophy. His genetic test and muscle biopsy revealed a diagnosis of DMD. The data of that time are no longer accessible due to distance in time.

A ten-day period of intermittent fever occurred in October 2018, when he was 18 years old. During the past 2 years, he suffered from liver disease, suspected brucella infection, and obesity. One year later, he fell ill with sepsis. Despite the fact that fevers had a fairly clear cause of infection, DMD had no concomitant symptoms except weakness of the lower limbs. Accordingly, systematic treatment hadn't been applied. At admission, the body temperature was 38°C, the heart rate was 90 beats/min, blood pressure was 110/72 mmHg, and breathing was 20 beats/min. Laboratory findings are as followed: creatine kinase (CK) 664 U/L, creatine kinase isoenzymes (CK-MB) 35.9 U/L, C-reaction protein (CRP) 29.66 mg/L, lactate dehydrogenase (LDH) 273 U/L, α-hydroxybutyrate dehydrogenase (αHBDH) 190 U/L, mycoplasma antibody: 1:160 positive. This patient was discharged after undergoing effective antiviral therapy (ganciclovir 0.25 g iv. Bid), mannatide (20 mg iv. Qd2) and creatine phosphate sodium (1g iv. Qd2). Because he did not have heart symptoms at that time, he did not have cardiac echocardiography test.

**Figure 1:**
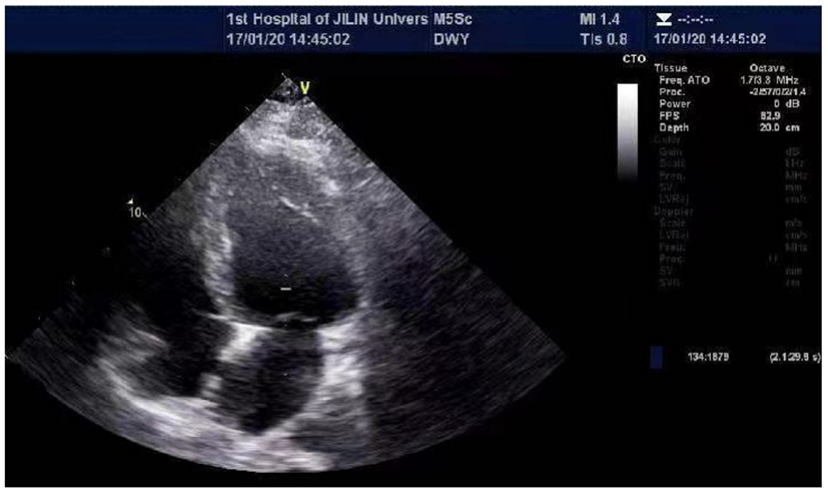
Echocardiographic of the patient's heart. The echocardiography revealed EF 29%, left atrial and left ventricle enlargement (left ventricular endomesosomal diameter 63 mm), reduced ventricular wall pulsation diffusion, decreased left ventricular systolic and diastolic function, and trace pericardial effusion.

He was admitted to the hospital with a high fever and numerous joint pains throughout his body for 4 days, with a temperature of up to 39°C in January 2020. At admission, his body temperature was 36.8°C, his heart rate was 84 beats per min, his blood pressure was 126/69 mmHg, and he was unable to walk upright. He is 175 cm tall and weighs 97 kg. His BMI is 31.67 kg/m2, which is overweight. Laboratory findings are as followed: CK 514U/L, CRP 43.41 mg/L, Interleukin-4 (IL-4) 3.42 pg/ml, IL-6 10.17 pg/ml, IL-10 8.13 pg/ml, tumor necrosis factor-α(TNF-α) 3.16 pg/ml. CT scan of the lungs revealed a small amount of inflammation in the left lower lobe and in the right lower lobe. After anti-infection treatment, he was discharged. After half a year, he was hospitalized again for a fever and muscle pain. Laboratory findings are as followed: CK 411 U/L, CK-MB 7.40 ng/ml, troponin I (Tn I) 0.067 ng/ml, N-terminal pro-B type natriuretic peptide (NT-pro BNP) 1250.0 pg/ml, CRP 45.67 mg/L, LDH 257U/L, mycoplasma antibody: positive. The lung CT was similar to the last one. In the abdominal CT, it was found that the patient had fatty liver, multiple enlarged lymph nodes around the abdominal aorta, and less muscle density on both sides of his back and buttocks, which should consider the possibility of fat infiltration. During echocardiography (Figure 1), the left ventricular ejection fraction (EF) was 39%, and left ventricle were enlarged (left ventricle endomesosomal diameter was 63 mm), abnormal ventricular movement, decreased left ventricular systolic and diastolic function, weak mitral regurgitation and trace pericardial effusion. The ECG showed sinus rhythm, a normal ECG. This time his cardiac function was level III and was placed on hormone therapy. He was discharged when his condition improved.

**Figure 2:**
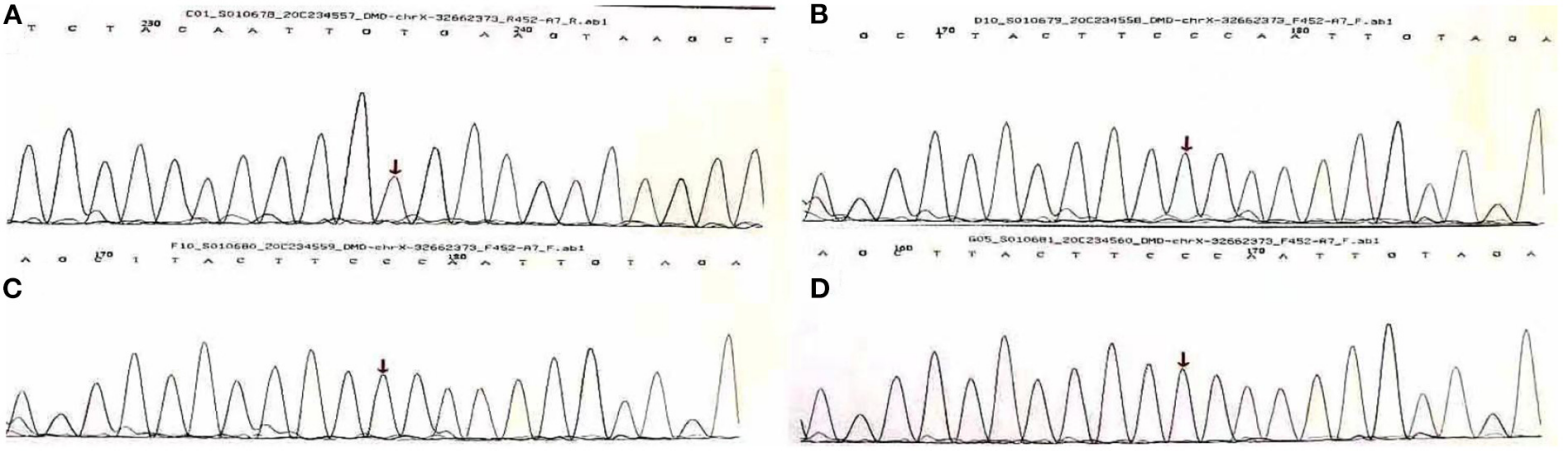
Gene sequencing results of Sanger method of Duchenne gene mutation in A family [Missense mutation occurred at the 1,207 base of the 11th exon of Duchenne gene in **(A)** (C.1207 G > T, P.G403x), arrows are the mutation sites, **(B–D)** are normal phenotypes].

In order to test if his heart disease was related to DMD, he and his family had another genetic test 2 months later. The results of sequencing suggested a nonsense mutation (c.1207G > T) in the exon eleven region of the DMD (Muscular dustrophy, Duchenne) gene, encoding amino acid p.G403X (guanine > thymine). It was found that neither his parents nor his sister carried heterozygous variation of this locus, and neither of them exhibited any clinical signs of muscular dystrophy (Figure 2).

In April 2021, again, he suffered from a fever and aching muscles. However, his heart function deteriorated. ECG showed left anterior branch block. The echocardiography revealed EF 29%, left atrial and left ventricle enlargement (left ventricular endomesosomal diameter 63 mm), reduced ventricular wall pulsation diffusion, decreased left ventricular systolic and diastolic function, and trace pericardial effusion. Diuretics, nitrates, and beta blockers are taken orally following discharge. His heart function was Grade III at that time. The hormone dose was reduced because his fever was controlled. Hormone should not be stopped immediately and should be reduced gradually. Even though he had another fever 8 months later, his heart function improved significantly, with his EF reaching 42%. The lung CT shows atrophy of the chest and back muscles and an increase in fat. His medication regimen was changed to oral beta blockers, coenzyme Q10, and hormones.

**Figure 3:**
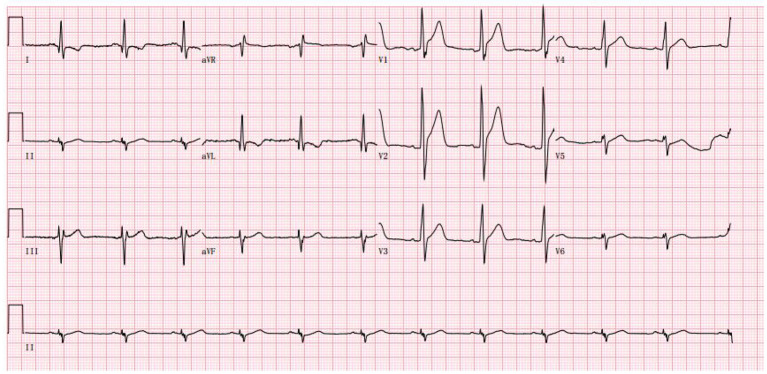
The abnormal ECG at the admission in June 2022 (Sinus rhythm and tall R wave in V1V2V3, left anterior branch block).

Palpitations began in June 2022, becoming more frequent over the following month. He developed acute myocardial injury. A rise of 0.18 ng/mL in TnI, a normal creatine kinase level, and 44% in EF were observed. The myocardial injury was persistent and troponin did not increase or decrease significantly. 24-h Holter electrocardiography showed a mean heart rate of 86 BPM, occasional premature ventricular contractions in pairs (673), occasional premature atrial contractions, and paroxysmal atrial tachycardia (total > 100 BPM, 283, maximum 6 min) (Figure 3). To treat the condition, the oral drugs were adjusted as hormone (in reduction), coenzyme Q10, trimetazidine and ARNI. Afterwards, the patient improved, and he was discharged (Table 1).

**Table 1:** The patient's timeline of admission, diagnosis and treatment.

**Symptoms**	**Age**	**Treatment**
Bilateral lower limb weakness	3 years old	
Intermittent fever for 10 days	18 years old	Antiviral
High fever and numerous joint pains throughout his body for 4 days	19 years old	Anti-infection
High fever and numerous joint pains throughout his body for 5 days	20 years old	Hormone therapy (oral 12 mg methylprednisolone twice a day)
High fever and numerous joint pains throughout his body for 4 days	20 years old and a half	Oral beta blockers, diuretics, nitrates, and hormones(16 mg methylprednisolone once a day)
High fever and numerous joint pains throughout his body for 4 days	21 years old	Oral beta blockers, coenzyme Q10, and hormones(16 mg methylprednisolone once a day)
Palpitations for 3 months	21 years old and a half	Hormone (5 mg methylprednisolone once a day), coenzyme Q10, trimetazidine, and ARNI

## Discussion and conclusions

DMD is a condition caused by a mutation in the dystrophin gene, which results in critical muscle wasting and death by age 30 (1). Children usually show symptoms in early childhood, and if untreated with corticosteroids by the age of 12 years, they will probably lose their abilities to walk (2). In the patient's case, his parents discovered that the patient had weakness in both lower limbs and could not walk independently at age 3, which coincided with DMD's symptoms.

DMD is a recessive autosomal-recessive disorder that results from mutations in the dystrophin gene that cause the absence of dystrophin protein (NIH U.S., 2020 National Library of Medicine) (1). The patient has a nonsense mutation (c.1207G > T) in exon eleven of the DMD (Muscular Dystrophy, Duchenne) gene, encoding amino acid p.G403X (guanine > thymine), which is one of the rarest type of genetic mutation. In the skeletal muscle cells, DMD encodes the dystrophin protein, which functions as a link between the intracellular cytoskeleton, the extracellular matrix, and the dystrophin–glycoprotein complex (2). Dystrophin-producing gene mutations cause a reduction in muscle ragility and result in contraction-induced injuries (5).

A diagnosis of DMD was determined based on clinical symptoms, age of onset, creatine kinase test, muscle biopsy analysis, and dystrophin mutation genetic testing (1). The current examination results show that the patient's chest, bilateral back, and gluteal floor muscles have been involved, which has resulted in fat infiltration and effected his overall motor function. Laboratory tests for patients with advanced DMD showed that creatine kinase levels were normal, which matched the patient's clinical findings.

Corticosteroids remain among one of the best methods for managing complications and have significantly increased the longevity of these patients (6). Evidence-based benefits of corticosteroids include a longer period of disease progression (7). Prednisone/prednisolone and deflazacort are the two most commonly used corticosteroids (7). Patients with DMD may benefit from taking 0.3 to 1.5 mg/kg/d of prednisone (8). In this case, the patient weighed 100 kg and took 5 mg of methylprednisolone orally daily, which is equivalent to 6.25 mg of ponisone, which is consistent with the dose required to benefit from ponisone treatment. He took corticosteroids as standard of care. Besides these medications, the FDA has approved other drugs as well, including eteplirsen (EXONDYS 51^®^), golodirsen (VYONDYS 53TM), and viltolarsen (VILTEPSO^®^) (1).

It is recommended to detect and treat cardiomyopathy in DMD patients early, as it can prolong survival (9). As the dystrophin protein is absent throughout life span, fibrofatty infiltration begins in the left ventricle's posterobasal wall (10). Infiltration of the ventricular wall can lead to fibrosis, thinning, dilation, and progressive decline of ejection fraction (11). According to the heart failure guidelines (12), this patient was Stage B HF. In spite of asymptomatic heart failure, there are structural and functional abnormalities that pose a greater risk for symptomatic heart failure (13). Yet for patients without myocardial infarction, there is no complete routine diagnosis or treatment. The pre-clinical stage of heart failure is treated with ARNI. ARNI is the abbreviation of Angiotensin receptor blocker Neprilysin. It is an anti-heart failure drug. After entering the body, it is hydrolyzed into valsartan and sacubitril. Valsartan is an ARB, which is a RAAS system inhibitor. Sacubitril can inhibit the degradation of various peptides including BNP and improve the level of endogenous BNP. Currently, in clinical guidelines (12), the efficacy evaluation of ARNI for heart failure with preserved ejection fraction and heart failure due to muscle metabolic diseases is limited. This patient's cardiac function improved after the application of ARNI, suggesting that such patients could benefit from the treatment of ARNI. The use of coenzyme Q10 and trimetazidine was also used to nourish the heart and improve its metabolism. The mechanism of trimetazidine is through inhibition of fatty acid β-oxidation. The metabolism of myocardial fat can be changed into oxidation using GLU, and the efficiency of oxygen utilization can be improved, so as to improve myocardial energy metabolism. The patient's palpitation symptoms improved after discharge, which indicated that the treatment was effective, but closer observation was still required.

DMD guidelines suggest baseline assessments of cardiac function consisting of an electrocardiogram, non-invasive imaging with echocardiography, or magnetic resonance imaging (CMR) at time of diagnosis (14). Individuals with asymptomatic disease should be re-examined annually (14). With progression of the disease, cardiac arrhythmia is more likely, which leads to further surveillance during the late ambulatory stage and the use of periodic 24-h Holter monitoring, as is directed by the specialist (1). Although ECG, echocardiography, and a 24-h holter ECG were performed on this patient, it was relatively late in the process. By the time myocardial involvement is detected, the ejection fraction has already decreased significantly and the myocardium has already been remodeled. As patients with DMD and other genetic metabolic diseases affecting the heart, we should remind them to regularly review electrocardiograms and echocardiograms. The prognosis of patients can greatly be improved by early diagnosis.

## Data availability statement

The original contributions presented in the study are included in the article/supplementary material, further inquiries can be directed to the corresponding author.

## Ethics statement

Written informed consent was obtained from the individual(s) for the publication of any potentially identifiable images or data included in this article.

## Author contributions

XL: methodology, investigation, formal analysis, and writing-original draft. WZhao: conceptualization, methodology, and visualization. SS: investigation, formal analysis, and writing-review and editing. WZhan: project administration and supervision. All authors contributed to the article and approved the submitted version.

## Conflict of interest

The authors declare that the research was conducted in the absence of any commercial or financial relationships that could be construed as a potential conflict of interest.

## Publisher's note

All claims expressed in this article are solely those of the authors and do not necessarily represent those of their affiliated organizations, or those of the publisher, the editors and the reviewers. Any product that may be evaluated in this article, or claim that may be made by its manufacturer, is not guaranteed or endorsed by the publisher.
